# Response to Comments on “Designing a Positive Health Dialogue Tool for Adolescents and Young Adults: A Mixed Methods Study”

**DOI:** 10.1111/hex.70159

**Published:** 2025-01-31

**Authors:** Marja van Vliet, Machteld Huber, Sigrid van der Zanden

**Affiliations:** ^1^ Institute for Positive Health Utrecht The Netherlands; ^2^ Public Health Service Brabant Zuid‐Oost Eindhoven The Netherlands

Dear Editor,

We appreciate the opportunity to address the concerns raised regarding our recent publication, “Designing a Positive Health Dialogue Tool for Adolescents and Young Adults: A Mixed Methods Study” (*Health Expect*. 2024 Oct;27(5):e70042). We value the constructive feedback and offer the following clarifications.

## 1 Sample Size and Generalisability

The quantitative aspect of our study involves a relatively small sample (*N* = 118), as acknowledged in the limitations section. These findings should, therefore, be interpreted with caution and are not generalisable to the entire population. Our main objective was to use this phase as an exploratory step, guiding the qualitative phase (focus groups and interviews), which forms the study's foundation. In qualitative research, a sample of *N* = 36 is not considered small; instead, the depth of data and achievement of saturation are critical indicators of validity.

Furthermore, we emphasise that this tool for adolescents and young adults builds upon rigorous research conducted during the development of both the adult and child versions. These earlier phases required distinct methodological considerations compared to starting from scratch.

## 2 Gender Imbalance and Demographic Focus

We acknowledge the gender imbalance within our sample (79% female). However, our recruitment strategy prioritised factors such as ‘having a disease’ and ‘educational level’, given their established influence on health perceptions, as reported in the literature (e.g., [1, 2]). Gender differences in this context tend to be less pronounced, as also noted in previous studies.

## 3 Lack of Long‐Term Assessment

We agree with the need to assess the long‐term effects of the dialogue tool, as highlighted in the discussion section. One of our aims in publishing this article was to encourage future studies evaluating the sustained influence of the tool, as has been done for the adult and child versions (e.g., [1, 3, 4]). Given the promising results of these earlier versions, we anticipate similar positive outcomes for this version.

## 4 Use of Self‐Reported Data

The dialogue tool is specifically designed to capture an individual's subjective experience, making self‐reported data the most appropriate method in this context. For evaluations requiring greater objectivity, we developed a separate measurement tool for the adult version [5, 6]. Developing a similar tool for adolescents and young adults would be a logical next step and is recommended in our discussion section. This will enable scientific evaluation purposes.

Nevertheless, the main goal of this dialogue tool is to promote a subjective understanding of personal health, providing a foundation for meaningful discussions about individual needs, priorities, and goals.

## 5 Discussion on Generalisability

We addressed concerns about generalisability in the methodological considerations section, specifically discussing its applicability across different cultural and socio‐economic backgrounds. This issue was also explored in our earlier rapid review [1]. With this specific version, we aim to contribute to the understanding of specificity for targeted groups. Further research is planned to test the dialogue tool across various groups to enhance understanding of the generalisability of the tool itself.

We trust these clarifications address the concerns raised and underscore our commitment to advancing research in this area.

## Author Contributions

**Marja van Vliet:** writing–original draft. **Machteld Huber** and **Sigrid van der Zanden:** review and editing.

## Conflicts of Interest

The authors declare no conflicts of interest.

6

## Data Availability

The authors have nothing to report.
